# Seizure (Ictal)—EEG Characteristics in Subgroups of Depressive Disorder in Patients Receiving Electroconvulsive Therapy (ECT)—A Preliminary Study and Multivariate Approach

**DOI:** 10.1155/2009/965209

**Published:** 2009-06-15

**Authors:** Björn Wahlund, Paolo Piazza, Dietrich von Rosen, Benny Liberg, Hans Liljenström

**Affiliations:** ^1^Department of Clinical Neuroscience, Division of Psychiatry, Karolinska Institutet, Karolinska University Hospital, Huddinge, 141 86 Stockholm, Sweden; ^2^Department of Energy and Engineering, Swedish University of Agricultural Sciences, 750 07 Uppsala, Sweden

## Abstract

*Objectives*. Examine frequency distributions of ictal EEG after ECT stimulation in diagnostic subgroups of depression. *Methods*. EEG registration was consecutively monitored in 33 patients after ECT stimulation. Patients were diagnosed according to DSM IV and subdivided into: (1) major depressive disorder with psychotic features (*n* = 7), (2) unipolar depression (*n* = 20), and (3) bipolar depression (*n* = 6). *Results*. Results indicate that the diagnostically subgroups differ in their ictal EEG frequency spectrumml: (1) psychotic depression has a high occurrence of delta and theta waves, (2) unipolar depression has high occurrence of delta, theta and gamma waves, and (3) bipolar depression has a high occurrence of gamma waves. A linear discriminant function separated the three clinical groups with an accuracy of 94%. *Conclusion*. Psychotic depressed patients differ from bipolar depression in their frequency based on probability distribution of ictal EEG. Psychotic depressed patients show more prominent slowing of EEG than nonpsychotic depressed patients. Thus the EEG results may be supportive in classifying subgroups of depression already at the start of the ECT treatment.

## 1. Introduction

Electroconvulsive therapy (ECT) is a well-established psychiatric treatment in which seizures are electrically induced in anesthetized patients for therapeutic effect mainly in various forms of mental depression. Electroencephalographic examination during seizures induced by ECT gives a very characteristic electroencephalogram (EEG) which however presents a large biological variation as well as nonstationarity patterns.

The EEG during seizures induced by ECT is very characteristic but shows a large biological variation as well as nonstationarity. Algorithm based in part of ictal EEG implemented in ECT equipment may be used to predict response of treatment and other potential clinical variables such as diagnosis [1, 2].

This study is a part of a larger project in which the characteristics of ictal EEG after ECT stimulation in affective disorders will be analyzed with various mathematical methods (traditional and new methods) in relation to clinical and other biological variables. 

 In EEG signals the classical statistical approach of interpreting measurement errors as generators of uncertainty is not valid. For EEG most of the noise is due to model error which cannot be considered to be random. We have not seen any EEG model where residuals are completely randomly distributed around a fitted model. This may be due to dependence structures, but it is not clear how to estimate this dependency in nonstationary series. Moreover, there are usually strong individual responses which nowadays usually are taken into account via random parameters, for example, in mixed models. However, in this report we stay with classical spectral analysis and will not present results where individual variation is incorporated.

Major depression is a heterogeneous condition, and the search for neural correlates specific to clinically defined subtypes has been inconclusive. Nevertheless, functional neuroimaging studies of major depression have identified neurophysiologic abnormalities in multiple areas of the orbital and medial prefrontal cortex, anterior cingulum, and related parts of the striatum and thalamus [3]. The abnormalities of function and structure in these cortical-subcortical circuits may be target for treatment with pharmacological and brain stimulation methods such as modern electroconvulsive therapy (ECT). 

The abnormality of the function in these cortical-subcortical circuits may depend on dysfunction in the major neuromodulating transmitters, imbalance in excitatory/inhibitory function, and lack of neurotrophic factors in turn causing decrease in synaptic plasticity [4–6].

Indication of ECT is pharmacological treatment resistant depression of various subtypes, that is, in this article referred to as psychotic depression, unipolar depression, and bipolar depression. In psychotic depression, patients experience delusions and/or hallucinations with colourings of low mood in addition to other depressive symptoms. In unipolar depression patients suffer from recurrent depressive episodes. In bipolar depression, patients suffer from a current depression that alternates with mania. 

ECT devices provide the opportunity to monitor induced seizures with electroencephalography (EEG), electrocardiogram, and electromyogram. EEG is a powerful method that registers cortical electrical activity and may be used to monitor the course of events in a seizure. 

In this treatment modality, electrodes are placed on the scalp, and a brief-pulse current is delivered, which depolarizes neurons, resulting in a generalized tonic-clonic seizure.

The course of the ictal EEG may be summarized as at least three events of distinct and sequential phase patterns. The first event contains high-voltage “sharp waves and spikes”, the second rhythmical “slow-waves”, and the third event an abrupt and well-defined ending, termed postictal suppression. This late phase is characterized by low amplitude and higher frequency [7].

Independent of electrode placement on the scalp has been shown to result in an increase in ictal slow wave delta power in the prefrontal cortex, with a stronger asymmetry seen with right unilateral ECT [8]. It has also been argued that when ECT occasionally induces an electrographic seizure with no overt convulsive activity, this is because seizures may be confined to nonmotor prefrontal regions. Thus, McNelly and Blumenfeld [9] showed that, for example, bifrontal ECT also resulted in focal activation, with maximal increases seen in the prefrontal and anterior cingulate cortex with a relative sparing of bilateral temporal regions

Most ECT studies have investigated the physiological mechanism of action in relation to clinical response [10]. To our knowledge this is the first study to report on subgrouping depressive disorder with the aid of seizure data obtained from ECT. 

The aim of this study was to analyze smooth density functions and time series of the seizure EEG, given in a clinical treatment setting, and relate these EEG characteristics to clinical subgroups of depressive disorder.

## 2. Subjects and Methods

### 2.1. Subjects

The research subjects consisted of 33 patients, 21 women and 12 men. Subjects were between the age 32 and 80. Two trained research raters used a structured interview (SCID-I, DSM-IV), 1994 [11] to establish a major depression diagnosis in all participants. Subdiagnoses were psychotic depression (*n* = 7), bipolar depression (*n* = 6) and patients with unipolar depressive episode (*n* = 20); see Table 1. All patients were clinically referred for unilateral ECT and were consecutively included in the study during a period of one year (February 2001–February 2002). All subjects were right handed and had not received ECT during the last three months. They had no evidence of neurological disease in their history, physical examination, or chart review. Subjects who were medicated with antidepressants and/or neuroleptics maintained their medication during the ECT series; see Table 2. Patients on lithium, benzodiazepine, or antiepileptics discontinued medication one week prior to ECT. 

### 2.2. Methods

#### 2.2.1. ECT Administration

All subjects received unilateral electrode placement according to the d'Elia method [12]. The patients received bidirectional pulse ECT (MECTA 5000Q ECT device; Mecta, Lake Oswego, OK). ECT was administered routinely three times a week (Monday, Wednesday, and Friday) for a period of 2–4 weeks. 

Two patients in the unipolar group were given thiopental sodium 1 mg/kg during anaesthesia. All others were given propofol 1.5–2.5 mg/kg. All patients received succinylcholin 1 mg/kg and ventilation until full saturation, that is, 100% oxygen by mask during anaesthesia.

The electrical stimulus intensity was determined by reference to a predefined chart, according to sex and age [13]. Pulse width (1 millisecond), stimulation time (6 seconds) and currency (800 mA) were fixed while stimulus frequencies varied.

#### 2.2.2. EEG Recording

Two channels of EEG were recorded using an MECTA 5000Q device. Electrodes were placed on the left and right prefrontal area and correspondingly reference electrodes on the left and right processus mastoideus. The channels over the prefrontal areas are called Fp1 and Fp2, according to the 10–20 system. Disposable stick-on Ag/AgCl electrodes with conductive gel were used (SLE diagnostics). Before application of electrodes the sites were cleansed with alcohol and an abrasive cleanser (NUPREP, SLE diagnostics) to ensure low electrode/scalp impedance. Registration of EEG was performed before, during, and after ECT. Registration ceased approximately 30 seconds after the beginning of the postictal period. By sampling at a frequency rate of 128 Hz, the continuous EEG signals were recorded. The data from the digital port of the MECTA equipment were simultaneously recorded, transferred to computer hard disc, and saved in files together with stimulus parameters.

#### 2.2.3. Artifacts of EEG

Artifacts were rejected manually; blind to subject and diagnosis by B.W. Movement artifacts were removed. All EEG recordings with alternating current (AC) disturbances were excluded from the analysis. When data was examined for artifacts, the definition of frequency bands was as follows: delta (0.5 < *P* ≤ 3.5 Hz), theta (3.5 < *P* ≤ 7.5 Hz), alpha (7.5 < *P* ≤ 12.5 Hz), beta 1 (12.5 < *P* ≤ 20.5 Hz), beta 2 (20.5 < *P* ≤ 32.5 Hz), and gamma >32.5 Hz. 

#### 2.2.4. Statistical and Mathematical Analysis

All calculations were performed on EEG signals collected directly after ECT stimulation and refer to the first ECT treatment. Collection was stopped 30 seconds postictally. EEG data can be difficult to interpret because there is no simple model for the dynamic variation of the signals. We have developed some specific statistical methods for the estimation which all are based on minor modifications of standard methods used in time series analysis and multivariate statistics [14]. Sampling the continuous EEG signals at a frequency rate of 128 Hz gave a Nyquist frequency of 64 Hz, that is, the highest frequency that can be estimated when studying the series in the frequency domain. The statistical analyses were mostly performed in the frequency domain using Matlab and the Signal Processing toolbox [15]. In particular, we addressed our attention on the estimation of the spectral density functions of the recorded time series of EEG [16–19]. 

The original EEG signals presented global as well as local trends. Therefore, in order to avoid any bias in the spectral estimates due to the presence of such trends the series were filtered by taking the first difference. After this preliminary operation, the series for each individual were Fourier transformed, and their spectral density functions were estimated.

## 3. Results

### 3.1. Analysis of Smooth Density Functions and Time Series

In this study we found identifiable and significant differences in the EEG characteristics, from Fp1 and Fp2, respectively, as well as the first treatment, between the different subgroups of patients with major depression. 

**Figure 1:**
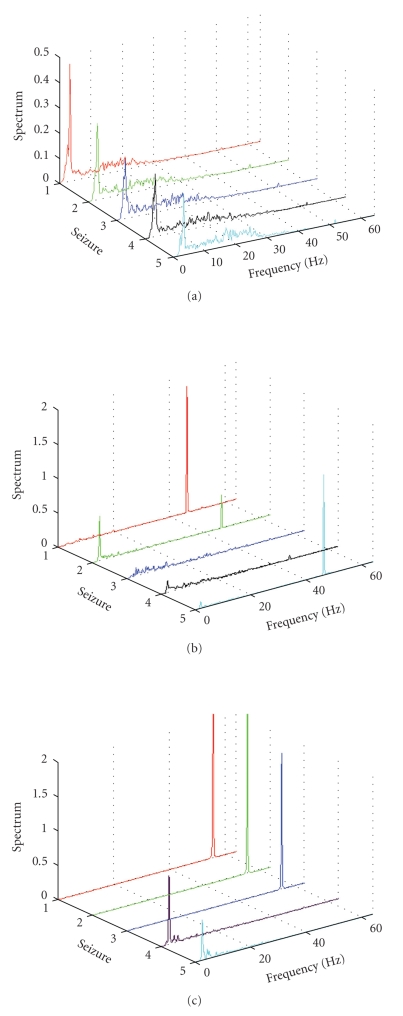
(a) Smoothed spectral density functions (sdfs) of channel Fp1 (Pat. 42—Psychotic depressed), (b) sdfs of channel Fp1 (Pat. 80—Unipolar depressed), and (c) Sdfs of channel Fp1 (Pat. 2—Bipolar depressed).

To illustrate how the smooth density functions are distributed for channel Fp1 of the first five seizures, we show the functions for one patient from each category, see Figures 1(a), 1(b), and 1(c), respectively. For statistical comparisons of the smooth density functions between the three clinical groups, please see Table 3.

In patients with psychotic depression we found a high occurrence of low frequencies in the EEG series. Most of the activity of the ictal EEG was found in the Delta and Theta frequency range (<10 Hz). In all spectra, the most prominent peaks were located in the Delta band. Moreover, the amplitude of such peaks tended to decrease over time, proportionally to the number of treatments received. All spectra are almost equal to zero for frequencies greater than 40 Hz, except for a small peak located at about 52 Hz (see Figure 1(a)).

In patients with unipolar depression, there was a more heterogeneous pattern in the frequencies of the ictal EEG. In almost all the seizures the highest peak of each smoothed spectral density function is located in the Gamma frequency range, more precisely around 47.3 Hz. However, considerable activity could be found in the Delta band where there were minor peaks, which were also characterized by decreasing amplitudes with increased number of treatments (see Figure 1(b)). Unipolar depression had more commonalties with bipolar depression than with psychotic depression, especially in the low-frequency domain of EEG.

In patients with bipolar depression we found a high occurrence of high frequencies. Almost all activity was found in the Gamma range where the global maximum of each spectrum (at about 55 Hz) is usually followed by two minor peaks at about 47 and 51 Hz (see Figure 1(c)). This frequency is found in the postictal-suppression phase of the seizure EEG. Moreover, the dominant activity as well as the most prominent peaks switches into the Delta range as the number of treatments increase. 

### 3.2. Classification

As a first step in finding out possible discriminatory variables between the three clinical groups, we stored the peak values of the spectral density functions of both prefrontal channels at 2.62, 2.87, 3.12, 3.25, 46.37, 46.62, 51.75, and 55.75 Hz. Thus, by selecting the most common frequencies from the EEG we totally had 16 observations, which came from both EEG channels.

**Figure 2:**
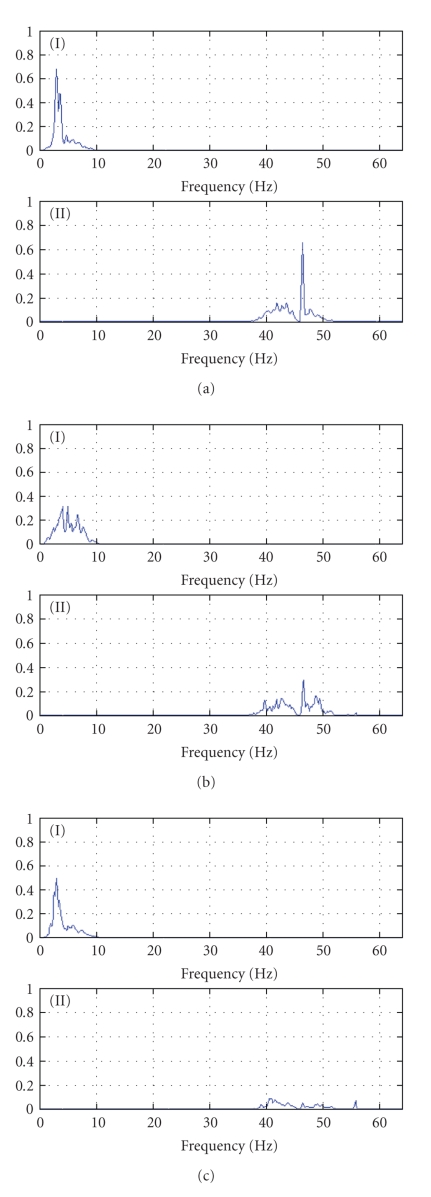
(a) Spectral density function of low-pass (I) and high-pass (II) series for a patient 1 (psychotic depression), (b) spectral density function of low-pass (I) and high-pass (II) series for a patient 19 (unipolar depression), and (c) spectral density function of low-pass (I) and high-pass (II) series for a patient 13 (bipolar depression).

Since these variables were highly correlated, we applied principal components analysis in order to avoid problems due to multicollinearity. The first six principal components were used in the multivariate analysis (see Figure 2), while they included more than 95% of the variability of variables from which they were originated. 

Sex, age, and medication had no statistical influence when performing analysis of covariance. 

Successively, in order to study in more detail the variation structure of the signals, we applied two different filters: a low-pass filter able to detect any possible difference within the Delta and Theta bands (0–8 Hz) and a high-pass filter in order to separate the Beta frequency band (more precisely the range 40–50 Hz). In both cases Yule-Walker filters of order eight were used.

Figures 2(a), 2(b), and 2(c)  show the smoothed spectral density functions for the low- and a high-pass-filtered series for one patient in each group. Concerning the low-pass series (graph at the top), it is very interesting to notice that many patients' spectra manifest, more or less, common characteristics. In each spectrum, in fact, a considerable percentage of the Delta activity (about 50%) is concentrated between 3 and 5 Hz. All the spectra have their greatest peak at 3.25 Hz, followed by a minor peak at 3.75 Hz. However there is also diversity in amplitude of such peaks, which tend to be higher in case of psychotic depression and smaller in case of unipolar depression. 

Finally, regarding the high-pass-filtered series (graph at the bottom), we can see that there actually are differences in the distribution of the variation of the processes among the different groups. Bipolar depressed patients display an EEG spectrum with a unique high peak of the smooth density function at 46.5 Hz while the remains of the function seem to be caused mainly by unspecific noise. This frequency is found in the postictal suppression phase of the seizure EEG.

Also unipolar depressed patients tend to have spectra with the main peak located at the same frequency but smaller in amplitude while bipolar depressed patients manifest their peak at 55.75 Hz even though their Gamma activity is quite uniform within the band. Hence we recorded, for both channels, the values of the spectra of the low-pass series at 3.25 Hz and those of the high-pass series at 46.5. These values were then transformed, via principal components analysis, into new uncorrelated variables.

The differences between the EEG characteristics of the psychotic depression patient group and patients with bipolar disorder were significant. Linear discriminant analysis based on principal components of the obtained frequencies in the EEG signals gave almost perfect separation between the individuals belonging to different subgroups of depression. The means, standard errors of means, and approximate confidence limits of the principal components analysis of the frequencies are shown in Table 3. 

Comparisons between the different smooth density functions (sdfs) of the clinical groups as well as the selected frequencies were tested pairwise. The first components of the eight selected frequencies from both channels separated the two clinical groups. Also the first and second principal components of the frequencies of the whole sdfs differed significantly between psychotic depression and bipolar disorder.

With repeated ECT the differences in EEG characteristics between the clinical subgroups were reduced. In data from the first ECT session, the clinical subgrouping matched the classification aided by EEG characteristics in 94% of the patients; see Table 4. However, cross validation showed that this matching is proportionally reduced with the number of treatments received (48.5% after the second seizure and 29.4% after the third). 

## 4. Discussion

There is a difference between EEG characteristics for patients with unipolar depression, psychotic depression, and bipolar depression. With repeated ECT these differences tend to even out. The ictal EEG of the first ECT is the most informative, that is, it has the highest capability of discriminating clinical subgroups. 

Several studies support a relationship between major depression with psychotic features and bipolar disorder [20–22]. The conversion rate from psychotic depression to bipolar disorder was 20% in these studies, and the age of those who converted was significantly lower than those who did not convert. In the present study the mean age of patients with psychotic depression was 50 years, and so it seems unlikely that there were any “latent bipolar” patients in the group of psychotic depressed patients. Discriminant analysis resulted in an almost perfect separation of the three clinical subgroups using high and low frequencies. However, our cross-validation analysis showed low predictability, and only a larger sample may provide a more reliable estimate of subgroup belonging. We are currently adding more individuals to our clinical database. 

There is considerable evidence from studies of different biological variables that point to distinct biological abnormalities in psychotic depression as compared with bipolar depression and unipolar depression [23]. The high remission rate (95%) after ECT in psychotic depressed patients as compared with depressed patients without psychotic features (83%) supports the argument that psychotic depression is a distinguishable nosological entity [24]. The EEG characteristics reported in our study agree in general with earlier reports [25–27]. 

This was an open study where treatment groups constitute of small and unequal sample sizes. Despite of no optimal study conditions, we saw significant differences between clinical subgroups. Many variables have been reported to influence EEG pattern. Patient medication and anaesthetic under treatment, patient age, ECT stimulus parameters, and the placement of EEG electrodes are all possible sources of variation. However, in our study, we saw no significant confounding of the results correlating with these variables, which may be due to the open study design. 

It may be argued that the number of EEG placements were too low to quantify EEG patterns related to ECT. The rational behind use of two EEG scalp electrodes was that this study was an open study, in ECT practice. Using two EEG electrodes is of course a limitation when studying EEG characteristics, such as topographic and spatial properties. However, the standard placements of EEG electrodes during ECT are Fp1 and Fp2. Data from these placements are included in the treatment algorithm implemented in the electrical device. The algorithm was based on ictal EEG data from 28 electrode scalp sites from over 200 patients and has been tested on data over 80 patients, confirming validity [28]. 

Data from Fp1 and Fp2 sites added most of the statistical variation to the regression model. Therefore, we believe that, for our purpose, the choice of these two sites was sufficient when studying seizure EEG characteristics in relation to various clinical variables.

 Another limitation in EEG studies to be considered is possible variation from EEG reference electrodes. We used standard ipsilateral electric references (i.e., bilateral processus mastoideus) according to Krystal et al. [28] when performing regression model. By careful placement of references electrodes as well as Fp1 and Fp2 electrodes, we believe that we minimized this source of variation.

Multivariate analysis on small sample size has disadvantages when many variables relatively to the independent experimental units are observed. Therefore, we applied variable reducing techniques (PCA) to make it possible to compare the clinical groups in a more effective way. The new variables (principal components) formed by PCA are asymptotically uncorrelated, and they may be included in a multiple regression analysis. We had some indications that observations from Fp2 differentiated the clinical groups PD and BD but not those from Fp1. This finding may indicate a difference between PD and BD in how the epileptic seizure propagates.

## 5. Conclusion

It is conceivable that the use of EEG data can aid diagnosis and leads to separate treatment for clinical subgroups of depressed patients. Classifying clinical subgroups can lead to better prophylaxis for the individual patient. During a 25-year period, 25% of unipolar depression will change diagnosis to bipolar disorder [29]. It is important to identify these individuals at an early stage of the disorder. With improved predictability, we can identify latent psychotic depression and bipolar depression (not yet diagnosed) in patients with unipolar depression using our method. The heterogeneous EEG characteristics for unipolar depression imply even more subgroups or features that may predict future succession in depressive disorders. The EEG of clinical subgroups becomes more alike with repeated ECT. The mechanism behind this observation point toward a late phase of divergent pathogenesis in depressive disorder. Moreover, this divergence may imply different endophenotypes in depressed individuals and is consistent with neo-Kraepelinian classifications of depressive disorder [30]. 

In this study EEG indicates to be an instrument for classifying subgroups of depression at initiation of ECT. However, the results should be replicated in larger studies.

## Figures and Tables

**Table 1:** Demographic variables of the subgroups of depression (psychotic depression (PD), Bipolar depression (BD), Unipolar depression (UD)): Sex (female (F), male (M)), Mean age ± standard error of mean (SEM), milli-Colomb (mC) ± SEM, and dynamical resistance (Ohm).

Group	Sex	Age	mC	Ohm
PD (*n* = 7)	F = 4, M = 3	55 ± 5	493 ± 47	235 ± 23
BD (*n* = 6)	F = 3, M = 3	56 ± 2	461 ± 42	248 ± 15
UD (*n* = 20)	F = 14, M = 6	53 ± 3	456 ± 32	251 ± 8

**Table 2:** Medication in individual patients. Labels: UD = unipolar depression; PD = unipolar depression with psychotic symptoms; BD = bipolar depression. Medication is indicated: 1 = with and 0 = without medication.

Patient ID	Group	Neuroleptics	Antidepressives
2	BD	1	0
13	BD	0	0
32	BD	0	0
121	BD	1	1
135	BD	0	0
146	BD	0	0

1	PD	0	0
15	PD	1	0
37	PD	0	0
41	PD	0	1
42	PD	0	0
122	PD	0	0
174	PD	0	0

8	UD	0	1
10	UD	0	0
16	UD	0	1
18	UD	0	0
19	UD	0	0
25	UD	0	1
27	UD	0	0
28	UD	0	0
30	UD	0	0
31	UD	0	0
34	UD	0	0
35	UD	0	0
39	UD	0	1
40	UD	0	0
45	UD	0	0
49	UD	0	0
74	UD	0	1
80	UD	0	0
97	UD	0	0
126	UD	0	1

**Table 3:** Mean values and standard error of mean (SEM) of the principal components (PC), summarizing spectrum of smooth density distribution of psychotic and bipolar depressed patients (PD and BD, resp.). Unipolar depressed (UD) patients cannot be differed from either psychotic or bipolar depressed patients. Fp2 denotes the left fronto-orbital EEG electrode.

Variable	Group	*n*	Mean	SEM
PC1	PD	7	−0.50***	0.65
of the whole	UD	20	−0.08	0.29
spectrum of sdf	BD	6	0.85	0.43

PC2	PD	7	0.70***	0.29
of the whole	UD	20	−0.15	0.32
spectrum of sdf	BD	6	−0.32	0.60

PC1	PD	7	1.13***	1.08
of selected	UD	20	0.02	0.53
frequencies	BD	6	−1.39	0.62

***Significant difference between PD versus BD, *P* < .001.

**Table 4:** Results of linear discriminant analysis using variables from the post ECT EEG frequencies of the 1st seizure. The correct number of number of individuals in respective subgroups of depressed (True group = patients) is on top of the table. The number of classified individuals according to the discriminant function is shown in the left margin (assigned to group = classified). Subgroups of depression: psychotic depression (PD), bipolar depression (BD), and unipolar depression (UD).

Assigned to group	True group			
	1	2	4	Total
PD	7	1	0	**8**
UD	0	18	0	**18**
BD	0	1	6	**7**

Total	**7**	**20**	**6**	**33**

*N* correct	7	18	6	

Proportion	**1**	**0.90**	**1.00**	
